# Cold bubble humidification of low-flow oxygen does not prevent acute changes in inflammation and oxidative stress at nasal mucosa

**DOI:** 10.1038/s41598-021-93837-x

**Published:** 2021-07-12

**Authors:** Lauriana Alves Santana, Suellen Karoline Moreira Bezerra, Beatriz Mangueira Saraiva-Romanholo, Wellington Pereira Yamaguti, Iolanda de Fátima Lopes Calvo Tibério, Tabata Maruyama dos Santos, Renato Fraga Righetti

**Affiliations:** 1 Serviço de Reabilitação, Hospital Sírio-Libanês, Rua Adma Jafet, 115 – Serviço de Reabilitação – 4º andar, São Paulo, SP 01308-050 Brazil; 2 Faculdade de Medicina FMUSP, Universidade de São Paulo, São Paulo, Brazil; 3 Public Employee of Sao Paulo Hospital (IAMSPE), São Paulo, Brazil; 4 University City of Sao Paulo (UNICID), São Paulo, Brazil

**Keywords:** Physiology, Health care

## Abstract

Some clinical situations require the use of oxygen therapy for a few hours without hypoxemia. However, there are no literature reports on the effects of acute oxygen therapy on the nasal mucosa. This study aimed to evaluate the acute effects of cold bubble humidification or dry oxygen on nasal Inflammation, oxidative stress, mucociliary clearance, and nasal symptoms. This is a randomized controlled cross-sectional study in which healthy subjects were randomly allocated into four groups: (1) CA + DRY (n = 8): individuals receiving dry compressed air; (2) OX + DRY (n = 8): individuals receiving dry oxygen therapy; (3) CA + HUMID (n = 7): individuals receiving cold bubbled humidified compressed air; (4) OX + HUMID (n = 8): individuals receiving cold bubbled humidified oxygen therapy. All groups received 3 L per minute (LPM) of the oxygen or compressed air for 1 h and were evaluated: total and differential cells in the nasal lavage fluid (NLF), exhaled nitric oxide (eNO), 8-iso-PGF2α levels, saccharin transit test, nasal symptoms, and humidity of nasal cannula and mucosa. Cold bubble humidification is not able to reduced nasal inflammation, eNO, oxidative stress, mucociliary clearance, and nasal mucosa moisture. However, subjects report improvement of nasal dryness symptoms (P < 0.05). In the conclusion, cold bubble humidification of low flow oxygen therapy via a nasal cannula did not produce any effect on the nasal mucosa and did not attenuate the oxidative stress caused by oxygen. However, it was able to improve nasal symptoms arising from the use of oxygen therapy.

## Introduction

Oxygen is a commonly used drug in the clinical setting and unquestionably saves lives^1^. Oxygen therapy is the administration of oxygen at concentrations greater than that in ambient air (20.9%) and has traditionally been delivered through nasal cannulas or simple face mask^2,3^.

Oxygen therapy is a fundamental therapeutic tool in the treatment of patients with acute and chronic respiratory failure, agitation, personality change, headache, nausea, increase in pulse and cyanosis^3,4^. It is considered to be of chronic use when used for 15 h or more during one day in chronic hypoxemic patients, very useful in terminally ill patients with cystic fibrosis and chronic obstructive pulmonary disease (COPD) and other populations^5^. However, during clinical practice it is common to use oxygen therapy for short-terms, even without hypoxemia, especially in the postoperative period, extubation, and anesthetic procedures^1,6^. The American Association For Respiratory Care^7^, based on some studies, recommends that nasal cannula oxygen delivered at flow rates ≤ 4 L/min need not be humidified, but these studies have subjectively evaluated patients’ dryness^8,9^.

Dry nasal supplemental oxygen is used clinically to prevent the infection and bacterial contamination of the bubble humidification reservoir^10,11^. However, the long-term inhalation of dry air may cause inflammation, mucociliary dysfunction, alterations in mucus properties and increase of the oxidative stress^12^. Studies investigating dry nasal supplemental oxygen on airway symptoms reported dryness in the mouth, nose, and trachea as well as headache and chest discomfort in healthy subjects^9,13^. The humidification was associated to some relief of nasal symptoms. However, all studies showed the long-term form of oxygen supplementation.

The aim of the present study was to investigate the acute effects of cold bubble humidification or dry supplementary oxygen on of the inflammatory and oxidative stress nasal responses, mucociliary clearance and nasal dryness symptoms in the healthy subjects.

## Results

**Figure 1:**
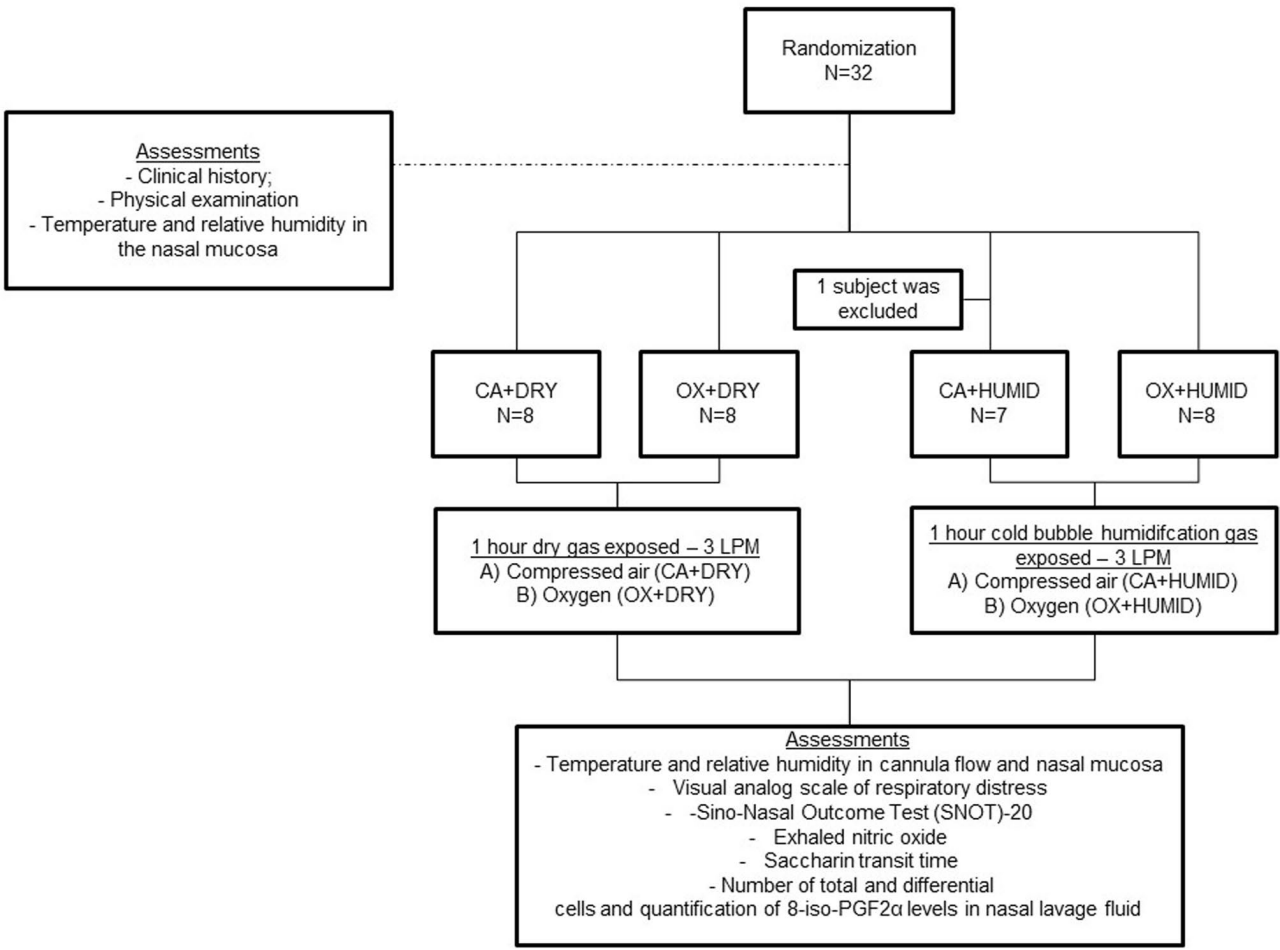
Study design.

Thirty-two healthy subjects were deemed eligible for study and were randomized to receive supplementary oxygen or compressed air at 3 LPM with or without humidification (Fig. 1). One subject was excluded from the CA + HUMID group after reporting nasal surgery. Table 1 shows that there were no differences among the groups in age, gender, BMI, pulse rate, body temperature, peripheral oxygen saturation and blood pressure. Table 1 also shows the medications used by each subject in the experimental groups.

**Table 1:** Characteristics of subjects.

Characteristic	CA + DRY (n = 8)	OX + DRY (n = 8)	CA + HUMID (n = 7)	OX + HUMID (n = 8)	P value
Age (year), mean ± SD	28.8 ± 6.7	27.0 ± 2.9	25.0 ± 1.6	25.8 ± 2.1	N.S.
Male, n (%)	2 (25%)	1 (12.5%)	0 (0%)	2 (25%)	N.S.
Female, n (%)	6 (75%)	7 (87.5%)	7 (100%)	6 (75%)	N.S.
BMI (kg/m^2^)	22.5 ± 2.2	23.3 ± 2,7	24.8 ± 7.3	27.1 ± 8.2	N.S.
Pulse rate (bpm)	74.7 ± 11.0	80.0 ± 10.1	75.7 ± 9.2	82.0 ± 13.9	N.S.
Body temperature (°C)	36.0 ± 0.4	35.9 ± 0.4	36.4 ± 0.2	35.9 ± 0.5	N.S.
Peripheral oxygen saturation (%), mean ± SD	98.7 ± 0.4	97.7 ± 1.0	98.5 ± 0.5	98.3 ± 0.5	N.S.
**Blood pressure**					
Systolic (mmHg), mean ± SD	108.7 ± 13.5	100.0 ± 0.0	111.5 ± 3.5	103.5 ± 11.8	N.S.
Diastolic (mmHg), mean ± SD	70.0 ± 8.6	67.5 ± 8.8	80.0 ± 0.0	75.0 ± 7.5	N.S.
**Medications, n (%)**					
Contraceptive	2 (25%)	3 (37.5%)	1 (12.5%)	0 (0%)	N.S.
Pantoprazole	1 (12.5%)	0 (0%)	0 (0%)	0 (0%)	N.S.
Sodium levothyroxine	1 (12.5%)	0 (0%)	1 (12.5%)	0 (0%)	N.S.
Clomipramine	1 (12.5%)	0 (0%)	0 (0%)	0 (0%)	N.S.
Finasterida	0 (0%)	1 (12.5%)	0 (0%)	1 (12.5%)	N.S.
Alprazolam	0 (0%)	1 (12.5%)	0 (0%)	0 (0%)	N.S.
Amitriptyline	0 (0%)	0 (0%)	1 (12.5%)	0 (0%)	N.S.
Trazodone	0 (0%)	0 (0%)	0 (0%)	1 (12.5%)	N.S.

### Temperature and relative humidity of the ambient gas flow at the outlet of the nasal cannula and the nasal mucosa

**Figure 2:**
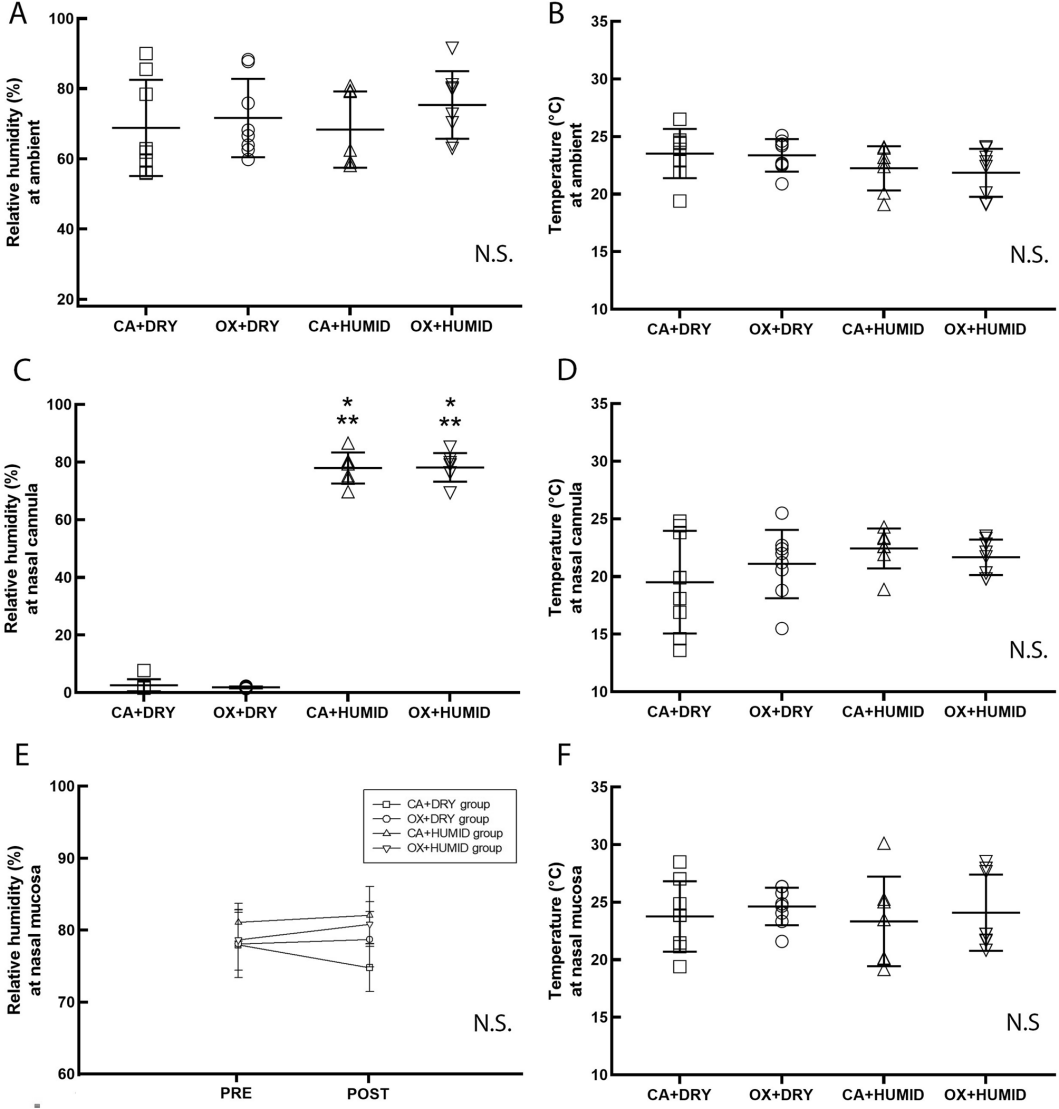
Relative humidity at ambient (**A**), temperature at ambient (**B**), relative humidity at nasal cannula (**C**), temperature at nasal cannula (**D**), relative humidity at nasal mucosa (**E**), and temperature at nasal mucosa (**F**). *P < 0.05 compared to CA + DRY group. **P < 0.05 compared to OX + DRY group.

Figure 2 shows the relative humidity (RH) at ambient (A), temperature at ambient (B), relative humidity at nasal cannula (C), temperature at nasal cannula (D), relative humidity at nasal mucosa (E), and temperature at nasal mucosa (F). There was no difference in the relative humidity of the ambient among the experimental groups. The CA + HUMID and OX + HUMID groups increased RH compared to CA + DRY and OX + DRY groups (P < 0.05). However, there was no difference in the RH between the before and after gas exposed at the nasal mucosa.

### Nasal symptoms

**Figure 3:**
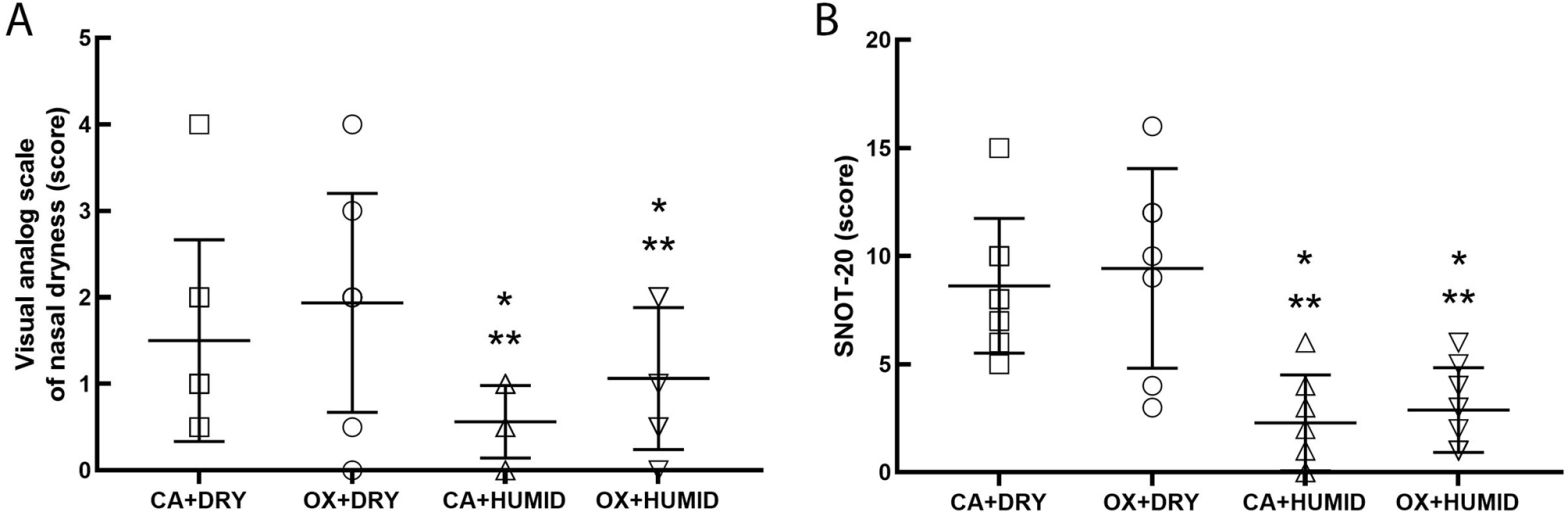
Visual analog scale of nasal dryness (**A**) and SNOT-20 (**B**). *P < 0.05 compared to CA + DRY group. **P < 0.05 compared to OX + DRY group.

Figure 3 shows the visual analog scale of nasal dryness (A) and SNOT-20 (B). Humidification in CA + HUMID and OX + HUMID groups reduced score in analog scale of nasal dryness and SNOT-20 compared to CA + DRY and OX + DRY groups (P < 0.05).

### Nasal inflammation

**Figure 4:**
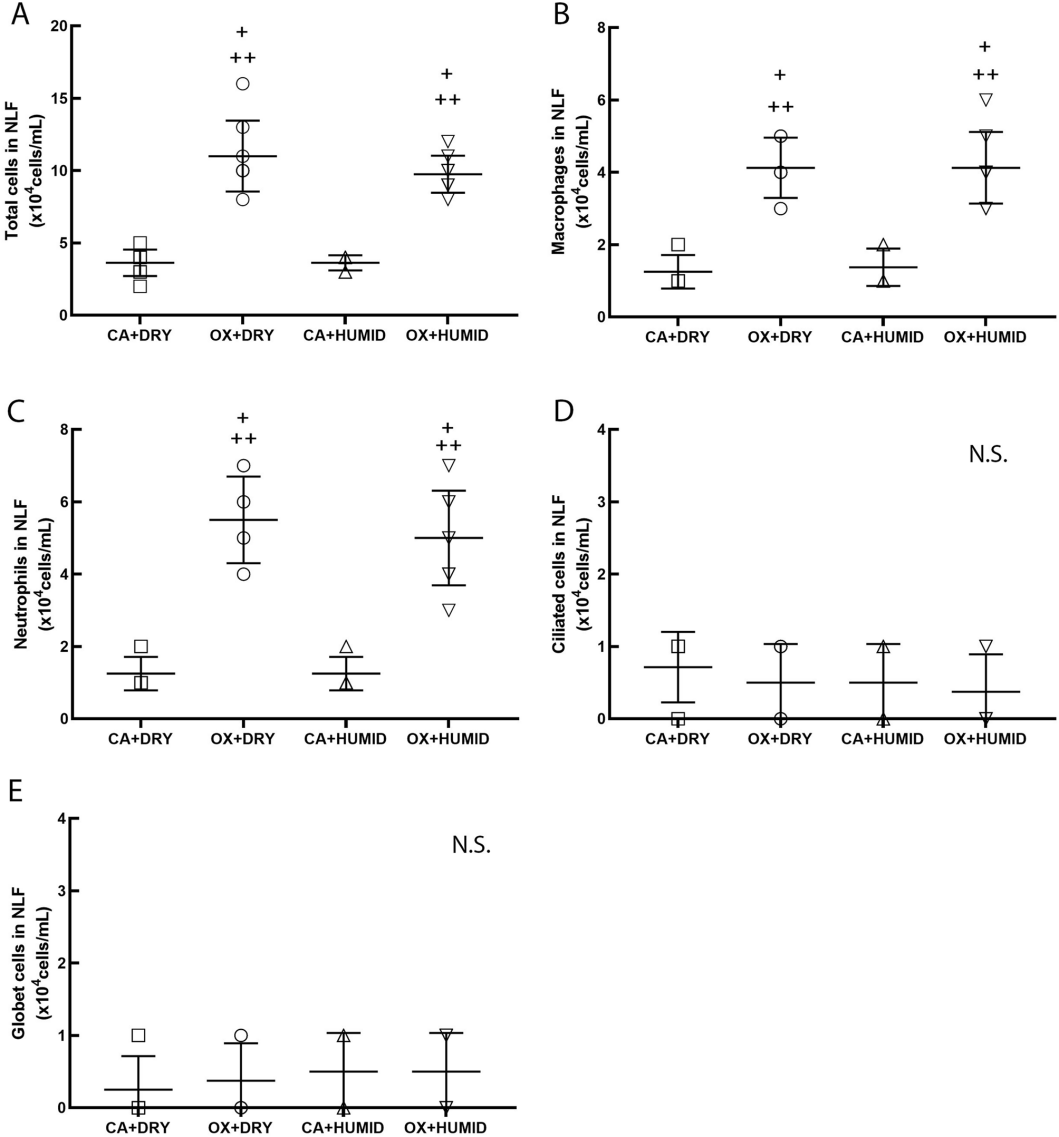
Nasal lavage fluid—total cells (**A**), macrophages (**B**), neutrophils (**C**), ciliated cells (**D**) and goblet cells (**E**). ^+^P < 0.05 compared to CA + DRY group. ^++^P < 0.05 compared to CA + HUMID group.

Figure 4 shows the total cells (A), macrophages (B), neutrophils (C), ciliated cells (D) and goblet cells (E). Oxygen supplementation increased the number of total cells, macrophages and neutrophils in the OX + DRY and OX + HUMID groups compared to CA + DRY and CA + HUMID groups (P < 0.05). There was no difference between OX + DRY and OX + HUMID groups.

### Oxidative stress evaluation

**Figure 5:**
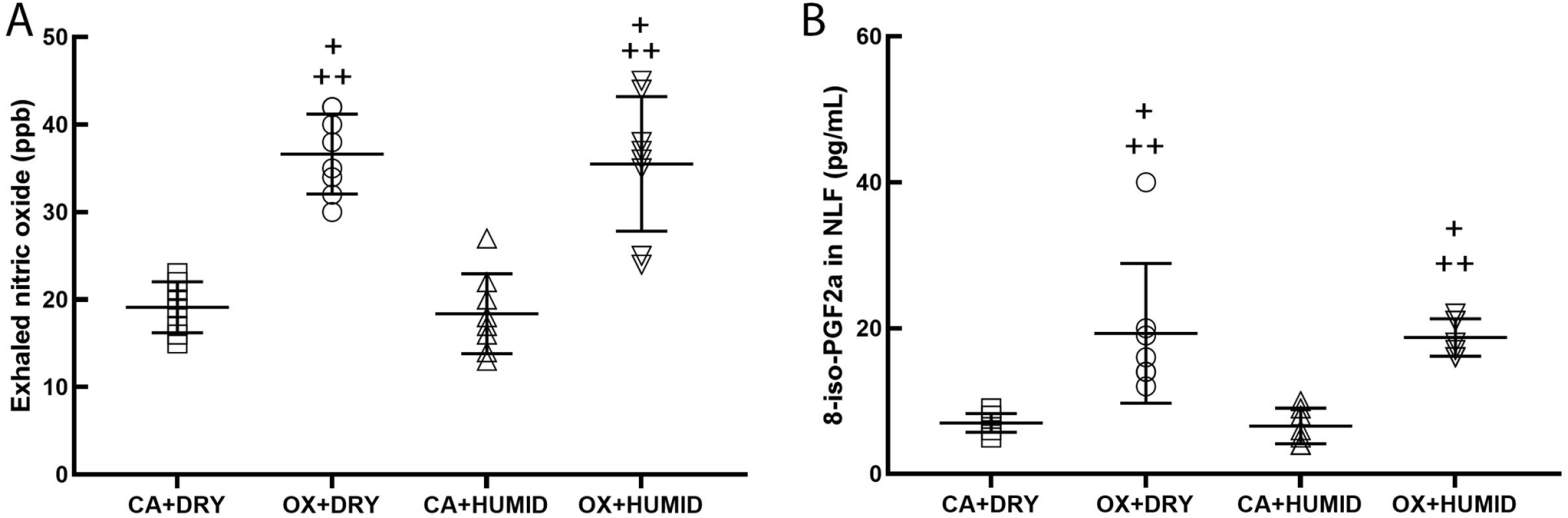
Exhaled nitric oxide (ppb) (**A**) and oxidative stress in the nasal lavage fluid (**B**). ^+^P < 0.05 compared to CA + DRY group. ^++^P < 0.05 compared to CA + HUMID group.

Figure 5 shows the exhaled nitric oxide (eNO) (A) and 8-iso-PGF2α (B) levels in supernatant of the NLF. OX + DRY and OX + HUMID groups showed increase in eNO and 8-iso-PGF2α levels compared to CA + DRY and CA + HUMID groups (P < 0.05). There was no difference between the OX + DRY and OX + HUMID groups.

### Saccharin transit time test

**Figure 6:**
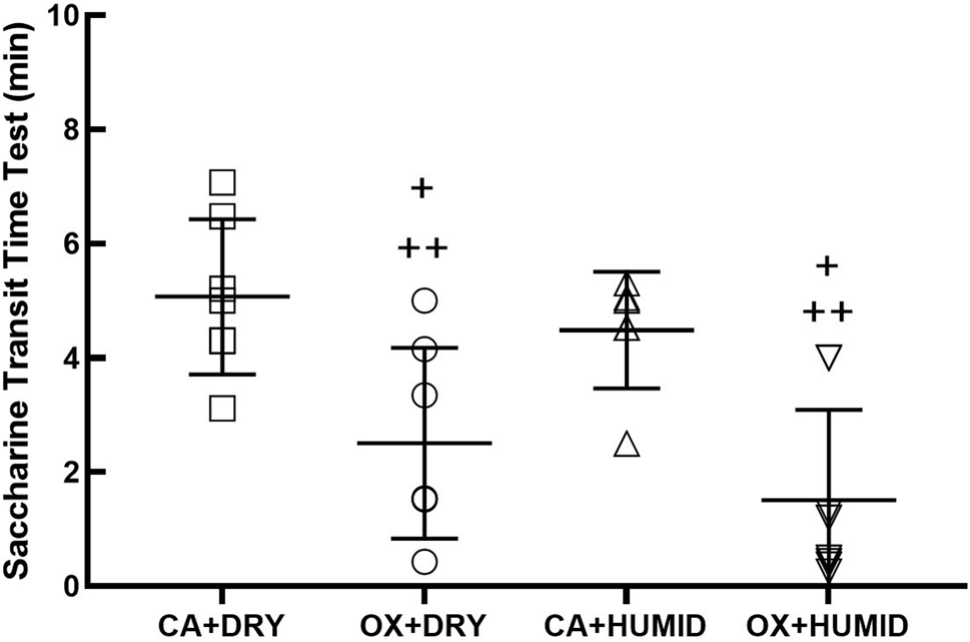
Saccharine transit time test (minutes). *P < 0.05 compared to CA + DRY and CA + HUMID groups. ^+^P < 0.05 compared to CA + DRY group. ^++^P < 0.05 compared to CA + HUMID group.

Figure 6 shows the STT results. OX + DRY and OX + HUMID groups reduced the STT compared to CA + DRY and CA + HUMID groups (P < 0.05). There was no difference between the OX + DRY and OX + HUMID groups.

### Pearson’s correlation

The Pearson’s correlation of STT with the measures for 8-iso-PGF2α levels, eNO, numbers of macrophages and neutrophils in NLF. Pearson’s correlation shows that there was a moderate negative correlation of STT with the levels of 8-iso-PGF2α (r =  − 0.60), eNO (r =  − 0.55) and the number of neutrophils (r =  − 0.51) in the NFL (P < 0.05). However, it showed a low negative correlation with the number of macrophages (r =  − 0.36) in the NFL (P < 0.05).

## Discussion

The present study evaluated the acute effects of the humidified and non-humidified oxygen therapy on the nasal mucosa with the use of the nasal cannula. We showed that oxygen humidification decreases nasal symptoms in subjects. However, it did not show a decrease in saccharin transport time, oxidative stress and inflammation in the nasal mucosa caused by acute oxygen exposure.

The cold bubble humidifier showed increased humidity of oxygen flow and compressed air, but did not show increased humidity in the nasal mucosa. Thus, this data showed that the humidified gas flow was not able to humidify the nasal mucosa. In accordance with ours results, Franchini et al.^12^ evaluated individuals diagnosed with chronic obstructive pulmonary disease on home oxygen therapy and showed that the humidified gas flow with nasal cannula with 3 LPM flow was not able to humidify the nasal mucosa.

Concerning inflammation, nasal lavage fluid analysis showed that oxygen supplementation increased the total number of cells, macrophages, and neutrophils in the oxygen-using groups (OX + DRY and OX + HUMID) compared to the groups using compressed air (CA + DRY and CA + HUMID), which showed us that oxygen, despite being used for a short period, was able to inflame the nasal mucosa. The OX + DRY and OX + HUMID groups showed increased numbers of neutrophils and macrophages in nasal lavage fluid. Neutrophils and macrophages are innate immunity cells responsible for the acute defense of the respiratory epithelium and for acute inflammation processes^14^. These results corroborate the study by Franchini et al.^12^, which showed that both the group of people who used humidified chronic oxygen and those who used the dry form had increased neutrophil, goblet cell and concentration ratios of epidermal growth factor (EGF). This allows us to realize that oxygen increases inflammation of the nasal mucosa in both acute and chronic use, regardless of the use of humidifiers. We also emphasize that in addition to their role in acute inflammation, neutrophils and macrophages are important triggers of the nitric oxide production process by augmentation of expression of inducible nitric oxide synthases^15–17^.

Related to the measurement of eNO, we observed that the groups that received oxygen (OX + DRY and OX + HUMID groups) showed an increased eNO compared to the groups that received compressed air (CA + DRY and CA + HUMID groups) and that there was no difference between the OX + DRY and OX + HUMID groups. This may be because in the respiratory tract NO is produced by a wide variety of cells, including epithelial cells, nerves, inflammatory cells (eosinophils, macrophages, neutrophils, and mast cells) and vascular endothelial cells^18,19^. Once produced, NO diffuses rapidly from the synthesis site, permeating cell membranes, and interacts with intracellular molecular sites^18^. NO is a highly reactive gas molecule. It is a free radical that reacts with other molecules such as oxygen, superoxide radicals or transition metals (such as those found within hemoproteins)^18^.

Still in the oxidative stress caused by oxygen, when evaluating the levels of 8-iso-PGF2α in nasal lavage we observed that the groups that received oxygen (OX + DRY and OX + HUMID groups) showed increased levels of 8-iso-PGF2α compared to the groups that received compressed air (CA + DRY and CA + HUMID groups), which shows us that increased oxygen exposure is capable of increasing oxidative stress. That happens because contact of oxidizing agents with the cell membrane leads to lipid peroxidation of the cell membrane, responsible for the formation of a series of prostaglandin-like bioactive compounds, known as isoprostanes^20^. Isoprostanes participate in different biological functions and are responsible for mediating certain aspects of oxidative injury^21^. Isoprostanes are produced via arachidonic acid peroxidation in a reaction catalyzed by free radicals and reactive oxygen species^22^. These components are useful biomarkers for oxidative stress, with increased isoprostanes in serum levels, urine, exhaled air, and bronchoalveolar lavage fluid^21,23^.

The isoprostane formation pathway is capable of producing 64 isomeric structures, of which 8-iso-PGF2α is the best characterized^24,25^. 8-iso-PGF2α is an isomer of PGF2α with contractile effects through binding to thromboxane A2 receptors in airway smooth muscle^26^. In addition, this agent causes increased pulmonary resistance, indicating that 8-iso-PGF2α via oxidative stress may be one of the mediators of pulmonary mechanical functional changes, although other mediators also perform this function^27^.

Several previous studies have reported that hyperoxia may increase oxidative stress in the lungs^28,29^. Recently, Phillips et al.^30^ described an increase in methylated alkane concentration in respiration in healthy volunteers after breathing 28% oxygen for 20 min. Carpagnano et al.^10^ showed that healthy individuals exposed to 28% oxygen therapy had increased 8-iso-PGF2α and interleukin (IL)-6 in the exhaled condensed air. Corroborating these findings, we now report increased levels of 8-iso-PGF2α and eNO in healthy individuals exposed to 3 LPM of oxygen, suggesting that supplemental oxygen may increase oxidative stress on the nasal mucosa and lungs. To confirm that oxygen exposure was responsible for this increase in oxidative stress, we administered compressed ambient air to healthy individuals and did not find increase in 8-iso-PGF2α and eNO levels. We also emphasize that humidification was not able to attenuate the 8-iso-PGF2α levels and eNO.

Regarding the STT, we observed that the groups that used oxygen had a significant reduction in saccharin transit time compared to the groups that used compressed air. This result differs from what Franchini et al.^12^ showed in patients with chronic obstructive pulmonary disease: that chronic use of oxygen worsens nasal mucociliary transport. This finding by the authors can be explained by the chronic response of neutrophil inflammation, as neutrophils during chronic inflammation increase mucus production by goblet cells, thus hindering mucociliary transport^31^. Salah et al.^32^ showed a decrease in mucociliary transport time in subjects receiving compressed air flows compared to subjects breathing in ambient air. Li et al.^33^ evaluated the role of nitric oxide in airway epithelial cell culture and showed that increased nitric oxide was able to increase the frequency of ciliary beating of the respiratory epithelium by stimulating guanosine ciliary monophosphate (cGMP) and the intracellular increase of Ca^+^ ion. In addition, studies show that increased oxidative stress is able to increase the activity of the Rho-kinase protein^34–36^. This protein performs several biological functions of cells, including controlling the cilia beat frequency in the respiratory epithelium^37,38^. Therefore, we believe that increased nitric oxide and 8-iso-PGF2α in the OX-DRY and OX-HUMID groups were a major factor in increasing mucociliary transport, reducing the response time of saccharin transport, and humidification was not able to alter this response. Furthermore, our correlation study showed a moderate negative relationship with the results of nitric oxide and 8-iso-PGF2α levels.

The scores on the visual analogue nasal dryness and SNOT-20 scale were significantly higher in the non-humidifying groups (CA-DRY and OX-DRY groups) than in the groups receiving cold bubble humidification, showing that the humidification of the flows allowed better comfort for the individuals. This reinforces what Miyamoto and Nishimura^13^ showed in their study, which exposed healthy individuals and individuals with some lung disease to varying flows and then subjectively assessed, through a visual analog scale, the perception of nasal dryness due to oxygen therapy and observed that in young healthy subjects the discomfort with flows was greater with non-humidified oxygen than with humidified oxygen at all flow rates and the discomfort difference between non-humidified and humidified oxygen is greater at flows above 3 LPM.

Our results show that humidification did not reduce the inflammatory process caused by oxygen and although the pathogenesis of oxygen damage is not well known, it is believed to be mediated by direct cell damage, activation of inflammatory cells and generation of reactive oxygen species^39^. It is generally accepted that increased production of reactive oxygen species plays an important role in triggering tissue damage from exposure to high oxygen concentrations^40^. Another interesting factor to note is that the humidification, despite reducing the discomfort of the subjects regarding the delivery of the flows, should be done with some care because Fauci et al.^11^ showed in their study, which evaluated bacterial colonization in reusable and non-reusable humidifiers, that reusable humidifiers promote bacterial growth. Therefore, when thinking about performing humidification to promote better comfort to individuals, one must take into consideration the type of device that will be used.

Franchini et al.^12^ studied the role of humidification in the chronic use of home oxygen therapy in inflammation, mucociliary transport and nasal symptoms in patients with chronic obstructive pulmonary disease. However, our study addressed the acute use of oxygen therapy, widely used in hospital clinical practice and emphasized the alterations of oxidative stress in the nasal mucosa.

This study has some limitations. The present study evaluated a single oxygen flow and a single device for oxygen therapy (nasal cannula). However, the oxygen therapy titration (3 LPM) used in the present study was based on the range recommended by British Thoracic Society guideline for oxygen use in adults in health and emergency settings for this device^3^. In addition, these results do not necessarily apply to patients receiving oxygen therapy with venturi mask, facial mask, or nasal high-flow oxygen, as these conditions were not studied.

## Conclusion

Cold bubble humidification of low flow oxygen therapy via a nasal cannula did not produce any effect on the nasal mucosa and did not attenuate the oxidative stress caused by oxygen. However, it was able to improve nasal symptoms arising from the use of oxygen therapy.

## Materials and methods

This cross-section, randomized and controlled study was approved by the Sírio-Libanês Hospital Institutional Ethical Committee for Research (number 3.346.094); it was performed in compliance with the Declaration of Helsinki. Subjects were entered into the study after written informed consent was obtained.

### Subjects

Thirty-two healthy subjects, female and male, aged > 18 years from the Hospital Sírio-Libanês from January 2019 through October 2019 participated in the study. Exclusion criteria were: chronic lung disease, hypoxemia, domiciliary long-term nasal low-flow oxygen, nasal surgery, use of nasal medications, diagnosis of acute or chronic rhinosinusitis, inability to taste saccharin, respiratory or other infections within 30 days of starting the study, pregnancy, and incapacity in understanding the protocol and experimental procedures. Subjects were oriented to avoid alcohol, coffee, and tea for 8 h before clinical assessments and all assessments were performed between 7:00 AM and 12:00 PM.

### Groups

The subjects were placed in a sitting position and we evaluated body temperature (°C), heart rate (bpm), peripheral oxygen saturation (%) and systolic and diastolic blood pressure (mmHg), weight (kg) and height (m) were evaluated. Then, the 32 subjects were randomized in four groups (8 each group):

(A) CA + DRY: individuals who received dry compressed air with nasal cannula;
(B) OX + DRY: individuals who received dry supplementary oxygen with nasal cannula;
(C) CA + HUMID: individuals who received cold bubbled humidified compressed air with nasal cannula;
(D) OX + HUMID: individuals who received cold bubbled humidified supplementary oxygen with nasal cannula.

### Gas exposure

For the gas exposure, the subjects were placed in a sitting position in the chair, with the upper limbs at rest and 90 degree flexion of the hips and knees. Subjects received supplementation of compressed air or oxygen through the nasal cannula (Lumiar HealthCare, Brazil). Supplementation might be dry or humidified by the cold bubble humidifier (AquaPak, Hudson RCI Oxygen Therapy Disposable Humidifier, CA, USA). The subjects received a gas flow of 3 L per minute (LPM) for 1 h. The individuals were told to breathe normally.

### Temperature and relative humidity of the ambient gas flow at the outlet of the nasal cannula and the nasal mucosa

The temperature in degree Celsius (°C) and percentage of relative humidity (%RH) of the ambient gas flow at the outlet of the nasal cannula and the nasal mucosa were collected. RH of the nasal mucosa was evaluated at the beginning of the experiment and after 1 h of gas exposure using the thermo-hygrometer (MTH-1380 Termopar K; Minipa)^12^. For this evaluation, the thermo-hygrometer sensor was positioned on the participant’s nose^12^.

### Nasal symptoms

After completing 1 h, the flow delivery was stopped and subjects rated their nasal dryness symptoms on the 10 cm visual analog scale of respiratory distress, marked from 0 to 4 quarters (0: none, 0.5: very mild, 1: mild, 2: moderate, 3: severe, 4: very severe) (Miyamoto et al., 2008). Nasal symptoms were evaluated by using the Sino-Nasal Outcome Test (SNOT)-20 questionnaires. Briefly, the SNOT-20 consists of 20 items in two major domains: upper airway symptoms (questions 1–10) and sleep quality (questions 11–20) each graded from zero to five (0 = no symptoms, 1 = minimal symptoms, 2 = small, 3 = moderate, 4 = serious, and 5 = the greatest symptoms possible). However, for the present study we used only the domain of upper airway symptoms, as it was the domain of acute effect. This questionnaire was previously adapted and validated in Portuguese^41,42^.

### Nasal inflammation

Nasal lavage fluid (NLF) was collected at each time point. Subjects were asked to tilt their head back at 30° and to close the nasopharynx with the soft palate. Five milliliters of room temperature isotonic sodium chloride solution (0.9% NaCl) was instilled into the left nostril. After 10 s, the subjects blew their nose forcefully into a sterile plastic container and fluid samples were centrifuged at 1800 rpm at 4 °C for 10 min. The cell pellets were resuspended in 1 mL of phosphate-buffered saline and 100 μL of each sample was centrifuged in a Cytospin for 6 min at 450 rpm. The total cell counts were performed using a *Neubauer* chamber and for differential cells counts. The slides were prepared and stained with Diff-Quick Reagent (Biochemical Sciences Inc., Swedesboro, NJ). 300 cells were counted per slide using an optical microscope, using the morphologic criteria^17,43^.

### Oxidative stress evaluation

The supernatant was separated from the pellet, transferred to sterile polypropylene tubes and stored at − 80 °C for the evaluation of 8-iso-PGF2α levels using ELISA kit in accordance with the manufacturer’s instructions (cod. CAYM-516351-480, Cayman Chemical, Ann Arbor, MI, USA).

For the eNO measurement, exhaled mouth air was filtrated before being collected in the Mylar bag, and the expiratory pressure achieved by the individual was monitored with a manometer. Subjects were advised to blow into a three Mylar bag, keeping the expiratory pressure of 12 cmH2O to avoid air contamination from the nasal cavity. All collected samples were evaluated for nitric oxide (NO) concentration by chemiluminescence (Sievers 280 NOA; Sievers Instruments, Boulder, CO). The equipment was calibrated before the start of each analysis. Results of NO concentrations were expressed in parts per billion (ppb)^43^.

### Saccharin transit time test

Nasal mucociliary clearance was measured by using the saccharin transit time test (STT). Subjects were seated and positioned with 10 degrees of neck extension. Granulated sodium saccharin (25 μg) was placed, under visual control, 2 cm inside the right nostril on the anterior portion of the middle turbinate. The time from particle placement until the first perception of a sweet taste in the mouth was recorded in minutes as measured with a digital chronometer. Individuals were instructed to maintain their initial position and not to perform deep or fast inhalation during the procedure, talk, cough, sneeze or sniff. If the sensation did not occur within 60 min, the test was stopped and the subject’s ability to perceive the taste of saccharin was verified by placing it on the tongue. Normal STT is < 12 min^44^.

### Statistical analyses

Data analyses were performed using the Sigma Plot 11.0 software version (Systat Software, SPSS Inc., USA). Results were presented as mean ± SD or proportions (%) when appropriate. Descriptive statistics were used to summarize subject demographic characteristics and *One-Way Analysis of Variance* (ANOVA) followed by the Holm–Sidak method for multiple comparisons and the χ^2^ test or Fisher’s exact test between patients with dry or humidification gases. We also obtained the Pearson’s correlation coefficient (R) to assess the associations of the saccharine transit test with the markers for oxidative stress and inflammatory cells. A P-value < 0.05 was considered statistically significant. For the total sample size we used the software G* Power version 3.9.1.2. The absolute nasal cannula humidification in the study of Franchini et al. was used as the main outcome, considering the following presupposition: effect size of 25%, α = 0.05, and a power of 0.80. The total sample consisted of 32 subjects.
